# IRMM-1000a and IRMM-1000b uranium reference materials certified for the production date. Part I: methodology, preparation and target characteristics

**DOI:** 10.1007/s10967-015-4227-x

**Published:** 2015-06-20

**Authors:** Zsolt Varga, Célia Venchiarutti, Adrian Nicholl, Judit Krajkó, Rožle Jakopič, Klaus Mayer, Stephan Richter, Yetunde Aregbe

**Affiliations:** European Commission, Joint Research Centre (JRC), Institute for Transuranium Elements, Postfach 2340, 76125 Karlsruhe, Germany; European Commission, Joint Research Centre (JRC), Institute for Reference Materials and Measurements, Retieseweg 111, 2440 Geel, Belgium

**Keywords:** Age dating, Radiochronometry, Uranium, Certified reference material, Nuclear safeguards and forensics

## Abstract

The paper describes the preparation and production of the reference materials, IRMM-1000a and IRMM-1000b, certified for the production date based on the ^230^Th/^234^U radiochronometer in compliance with ISO Guide 34:2009. The production date of the reference materials corresponds to the last separation of ^230^Th from ^234^U, i.e. when the initial daughter nuclide content in the material was finally removed. For the preparation low-enriched uranium was used, which was purified using a unique methodology to guarantee high U recovery and Th separation efficiency. The CRM is intended for calibration, quality control, and assessment of method performance in nuclear forensics and safeguards.

## Introduction

In order to avoid the malicious use of nuclear materials, an international safeguards system directed by the International Atomic Energy Agency (IAEA) has been set up to verify the correctness and completeness of states’ declarations about the nuclear-related activities and nuclear material accountancy [1]. However, if such materials are diverted and afterwards interdicted, detailed investigation is required to identify the possible origin, intended use and hazard related to the material. Such analyses, which have recently evolved to a new discipline called *nuclear forensics*, involve comprehensive physical, chemical and isotopic analyses (e.g. physical dimensions, crystal structure, radioactive and stable chemical impurities) as well as the interpretation of the measured data along with additional information on the material in question (such as open-source information or data from the law enforcement authorities) [2–4]. Several characteristics (so-called *signatures*) of the material can be used for such purpose, such as isotopic composition of U, Pb or Sr, elemental impurities, trace-level radionuclide content, crystal structure or anionic residues [5–11]. Besides these parameters, the elapsed time since the last chemical purification of the material (commonly referred to as the “age” of the material) can also be measured for radioactive and nuclear materials [12–18]. This unique possibility is based on exploiting the presence and decay of radionuclides: during its production, the radioactive material is chemically purified from the impurities including also the radioactive decay products. After the separation, the radioactive progenies start to grow-in into the material. Assuming that the parent-daughter separation was complete during the chemical processing, by the measurement of the daughter-to-parent ratio in the sample (often referred to as chronometer), the elapsed time since the last separation can be calculated according to the decay equations. This age and the respective production date can help either to identify the origin of the questioned unknown sample or to verify the source of the feed (starting) nuclear material used for production. In contrast to most other characteristics used in nuclear safeguards and forensics, the production date of the material is a predictive signature, thus it does not require comparison samples for origin assessment (i.e. a self-explaining parameter). This feature makes the production date one of the most prominent signatures for attribution.

The nuclear forensic findings should not be scientifically or judiciary questionable during the security response or the prosecution process. Although the quality assurance in nuclear forensic investigations is of primary importance, currently no uranium reference material (RM) with certified production date is available to assure the confidence in the quality of results for the age measurement in nuclear forensics [19]. The important and emerging need for such materials have been recently expressed by the community involved in national or international security programs [20, 21]. In practice, due to the lack of radiochronology RMs, reference materials certified only for major radionuclide composition are used to check the accuracy of the results by comparing them with the final purification dates from archives of these materials (usually referred to as *assumed, model* or *archive* ages) [14, 22, 23]. Additionally, since ^230^Th is present at trace-level in the nuclear materials (typically in 10^−10^–10^−7^ g per gram sample depending on the enrichment and age), the Th/U measurements are still challenging for most nuclear forensic laboratories despite of the availability of state-of-the-art analytical techniques. This was also demonstrated during a recent material exchange exercise of the Nuclear Forensics International Technical Working Group (ITWG) [24]. Therefore, assigning a consensus or agreed value for a uranium age dating certified reference material by an inter-laboratory comparison bears the risk that proper accuracy and traceability of this value cannot be achieved. Furthermore it would be a lengthy exercise and requires combining results which could have a significant bias and large uncertainties.

In this context, our objective was the preparation of a uranium age dating reference material (CRM) certified for production date, which can be applied for the validation of age dating measurements based on the ^230^Th/^234^U chronometer. The preparation of the material and the forthcoming measurements were performed at the European Commission Joint Research Centre Institute for Transuranium Elements (EC-JRC-ITU), while the documentation, inter-laboratory comparison and certification were managed by the European Commission Joint Research Centre Institute for Reference Materials and Measurements (EC-JRC-IRMM in compliance with the ISO Guide 34:2009 [25]. Prior to its release as CRM, the produced reference material was subjected to an EC-JRC-IRMM inter-laboratory comparison, REIMEP-22 (Regular European Inter-Laboratory Measurement Evaluation Programme) on “U Age-dating– determination of the production date of a uranium certified test sample”, carried out according to ISO 17043:2010 [26]. The material was produced (purified) under well-known and well-defined conditions assuring that the last chemical separation (production date) of the material is known, and the residual ^230^Th is negligible. Any residual ^230^Th present after the production would result in a systematic bias, which has to be measured and taken into account for the calculation of the certified production date. If the residual ^230^Th is confirmed to be negligible, the ^230^Th present in the material will solely come from the ^234^U decay and its amount depends only on the radioactive decay laws. The advantage of this approach is the accurately and very precisely known ^230^Th/^234^U amount ratio as a function of time, which is far more suitable for a reference material compared to what is achievable by only using the current measurement capabilities. The material preparation is based on the methodology developed previously [27, 28]. The Th separation efficiency, which is the key element to make sure that no residual ^230^Th remains after the preparation and the ^230^Th/^234^U ratio is governed only by the ^234^U decay, was verified by three independent methods. Although the primary objective was to certify the production date using the ^230^Th/^234^U chronometer, the separation was performed in such a way that ^231^Pa was also removed from the sample, so that the material could be applicable for the ^231^Pa/^235^U chronometer as well. However, in the lack of an appropriate tracer and since the Pa separation could not be monitored by gamma spectrometry, the material was certified only for the ^230^Th/^234^U chronometer.

Note that this age dating reference material certification is very unique, since the aim was to certify the production date of the material based on the ^230^Th/^234^U ratio. However, this ratio is continuously increasing due to the ingrowth of the daughter nuclide. Therefore, a specific approach compliant with ISO Guide 34:2009 was needed for certification of this CRM. The certified measurand of IRMM-1000a and IRMM-1000b is thus not the age derived from the ^230^Th/^234^U ratio itself, as in other RMs used in age-dating, but the date when this material was produced with negligible Th decay product present at that time.

## Calculation of the age of the sample

Production date (age) determination of uranium materials is most often carried out by the ^230^Th/^234^U chronometer. This age dating is based on the decay of the relatively long-lived ^234^U (*T*_1/2_ = 245,250 ± 490 years) to ^230^Th (*T*_1/2_ = 75,690 ± 230 years) and the disequilibrium between these two radionuclides [15, 29]. After the last chemical separation of ^234^U during the preparation of the nuclear material, the concentration of the ^230^Th daughter nuclide is continuously increasing in the uranium-containing material. The theoretical ^230^Th amount formed by the decay can be calculated applying the equations for radioactive decays as follows:

1 $$ \frac{{N_{\text{Th-230}} }}{{N_{\text{U-234}} }} = \frac{{\lambda_{\text{U-234}} }}{{\lambda_{\text{Th-230}} - \lambda_{\text{U-234}} }}\left( {e^{{ - \lambda_{\text{U-234}} t}} - e^{{ - \lambda_{\text{Th-230}} t}} } \right) + \frac{{N_{\text{Th-230}}^{ 0} }}{{N_{\text{U-234}} }}e^{{ - \lambda_{\text{Th-230}} t}} $$

where *N*_Th-230_*/N*_U-234_ is the amount (number of atom) ratio in the sample, *λ*_Th-230_ and *λ*_U-234_ are the decay constants of ^230^Th and ^234^U, respectively, *N*_Th-230_^0^ is the residual ^230^Th after the chemical separation, and *t* is the elapsed time since the separation of the radionuclides. Age dating models assume that the sample behaves as a closed system, meaning that there is no loss or increase for either the ^234^U parent nuclide or for the ^230^Th decay product. If the initial concentration of the daughter nuclide is zero after the last chemical separation (i.e. the separation was complete, *N*_Th-230_^0^ equals to zero), and the atom ratio of ^230^Th and ^234^U is measured, the elapsed time, i.e. age of the sample (*t*) can be calculated as follows:

2 $$ t = \frac{1}{{\lambda_{\text{U-234}} - \lambda_{\text{Th-230}} }}\ln \left( {1 - \frac{{N_{\text{Th-230}} }}{{N_{\text{U-234}} }} \times \frac{{\lambda_{\text{Th-230}} - \lambda_{\text{U-234}} }}{{\lambda_{\text{U-234}} }}} \right) $$

However, as the ^230^Th/^234^U method is highly sensitive to the initial purity of the material, a very high degree of separation (more than 10^7^) has to be achieved for this chronometer to eliminate the positive bias caused by residual ^230^Th in the material (i.e. incomplete zeroing). This high separation factor was not accomplished in the past for several uranium isotopic standards, and a discrepancy was found between the measured production dates and the known archive date of the material preparation [23]. Therefore, re-certification of already available uranium isotopic standards for their production dates based on the ^230^Th/^234^U can be problematic due to the disagreement of the measured (model) age and the actual real production date.

## Target characteristics of the certified reference material

Based on the previous ITWG material exchange exercise, the following prerequisites for the CRM production in compliance with ISO Guide 34:2009 and target criteria of the final material were defined:

1. In order to assure that the production date based on the ^230^Th/^234^U chronometer agrees with the date of the last chemical separation, the residual ^230^Th at the time of final separation has to be negligible (Eq. 2). In order to have less than 6 h’ bias coming from the residual ^230^Th, the ^230^Th/^234^U amount (atom) ratio at the time of the separation has to be less than 1.9 × 10^−9^.
2. Low-enriched uranium is the most suitable starting material with a relative mass fraction *m*(^235^U)/*m*(U) below 5 %). It is one of the most often occurring types of illicit nuclear materials. Moreover, the lower enrichment also eases international transport.
3. Two different unit sizes should be produced: 20 mg U-containing unit intended for mass spectrometric analysis and 50 mg U-containing unit for radiometric techniques.
4. At least 5 grams of purified uranium has to be prepared in order to produce more than 150 units (about 110 units of 20 mg U and about 55 units of 50 mg U). This indicative number of units includes the units required for the certification measurements (value assignment, confirmation, homogeneity and stability measurements) and for the REIMEP-22 inter-laboratory comparison.
5. The final material has to be kept in solid form to avoid possible loss and adsorption of ^230^Th [27].
6. The CRM should be made available to the laboratories a few months’ after preparation, when the purified material contains well measurable amount of ^230^Th daughter product and is fit-for-purpose for most laboratories.

## Experimental

### Reagents and materials

All labware was thoroughly cleaned before use. Suprapur grade hydrofluoric and nitric acids (Merck, Darmstadt, Germany) were used for the sample preparation. HNO_3_ was further purified by subboiled distillation (AHF analysentechnik AG, Germany). For dilutions ultrapure water was used (Elga LabWater, Celle, Germany). A ^233^U isotopic standard was used to spike the samples for the uranium concentration measurements. The ^233^U concentration in the spike was calibrated against EC NRM 101 uranium metal by thermal ionization mass spectrometry (TIMS). A custom-made natural Th-solution from Spex Certiprep Inc. (Metuchen, USA) at a Th concentration of 1000 μg g^−1^ was used for the ^230^Th isotope dilution measurements as spike, and this standard was also added to the material after the first separation step to verify the Th separation efficiency. The relative expanded uncertainty of the ^232^Th concentration in the standard is 0.5 % (*k* = 2), and its *n*(^230^Th)/*n*(^232^Th) ratio is (4.76 ± 0.28) × 10^−6^). Nominally 1 % enriched uranium U-010 standard reference material from National Bureau of Standards (USA) was used to correct for instrumental mass discrimination. The certified isotope reference materials IRMM-035 (certified *n*(^230^Th)/*n*(^232^Th) is (1.1481 ± 0.0078) × 10^−5^) and IRMM-185 (certified *n*(^235^U)/*n*(^238^U) is (2.00552 ± 0.00060) × 10^−2^) were used to check the accuracy of the thorium and uranium isotope ratio measurements, respectively.

TEVA extraction chromatographic resin (50–100 μm particle size, active component: aliphatic quaternary amine) supplied by Triskem International (Bruz, France) was used for the thorium separations for both the age dating measurements and production of the certified reference material. For the preparation of the certified reference material 1.6 mL of the TEVA resin and 0.1 mL silica gel (10–40 μm particle size, purified, Merck) were placed in plastic Bio-Rad holders (diameter: 6 mm, length: 30 mm) in a “sandwich” arrangement separated and covered by porous Teflon frits to avoid mixing (Reichelt Chemietechnik Heidelberg, Germany). Before use, the resin was cleaned with 1 mL of 0.02 M HF/0.02 M HNO_3_ followed by conditioning with 3 mL 2 M HNO_3_. The ^230^Th separation and measurement for the age dating is discussed in detail elsewhere [27].

### Starting uranium material

An appropriate aliquot of low-enriched uranium (approximately 3.6 % ^235^U) was identified at EC-JRC-ITU at sufficiently large quantity as a feed solution for the CRM production. The low-enriched uranium feed solution was prepared by the dissolving and mixing of high-purity natural and low-enriched uranium dioxide pellets of three different origins. The UO_2_ pellets were dissolved in 8 M HNO_3_ in Teflon Erlenmeyer flasks while heating to about 100 °C overnight. From this solution an aliquot solution containing about 6 g of uranium was prepared for the separation in 3 M HNO_3_. The exact isotopic composition of the material given as isotope mass fractions (%) is ^233^U: <2 × 10^−6^, ^234^U: 0.028541 ± 0.000037, ^235^U: 3.6066 ± 0.0034, ^236^U: 0.09000 ± 0.00014, ^238^U: 96.275 ± 0.040. The model age of this uranium solution was 12.04 ± 0.23 years (reference date: 12 July 2012), measured by the EC-JRC-ITU age dating procedure [27], corresponding to a ^230^Th/^234^U amount ratio of 3.40 × 10^−5^. This implies that during the CRM preparation the Th separation factor (quotient of the Th/U ratio in the initial material and in the final reference material after the chemical separation from U) will have to be higher than 1.76 × 10^4^ in order to have less than 6 h’ bias from the residual ^230^Th in the final purified material. The total Th content in the material is 0.049 ± 0.006 μg g^−1^ U, measured by the EC-JRC-ITU Analytical Services.

### Instrumentation and analytical measurements

The U and Th isotopic analyses, the U, Th and impurity concentration measurements were carried out using a double-focusing magnetic sector inductively coupled plasma mass spectrometer (ICP-MS) equipped with a single electron multiplier (Element2, Thermo Electron Corp., Bremen, Germany). All measurements were carried out in low resolution mode (*R* = 300) using a low-flow micro-concentric nebulizer operated in a self-aspirating mode (flow rate was approximately 50 μL min^−1^) in combination with a Teflon Scott-type spray chamber. Concentrations of isotopes of interest necessary for the production date calculation were experimentally determined as a function of ^230^Th/^232^Th and ^234^U/^233^U ratios according to the isotope dilution method (IDMS). The measured amount contents of ^230^Th and ^234^U measured by IDMS were used to calculate the (model) age of the material according to Eq. (2). The measured isotope ratios obtained by ICP-MS were corrected for instrumental mass bias using linear correction [30]. The U concentrations and isotopic compositions were also measured by TIMS using a MAT261 (Finnigan MAT, Bremen Germany, for U isotopics and concentration) and a Triton (Thermo Scientific, Bremen, Germany, for U isotopics, measured by the modified total evaporation method [31]) instruments by the EC-JRC-ITU Analytical services.

Impurity measurement of the purified uranium solution was performed using the Element2 ICP-MS. A sample aliquot was diluted to about 100 μg U g^−1^ concentration gravimetrically, and measured using Rh internal standard with matrix-matched calibration [32].

The gamma spectrometric measurements were performed using a well-type HPGe detector (GCW 2022 model, Canberra Industries Inc., USA) with approximately 20 % relative efficiency and a resolution of <1.7 keV at 185.6 keV. The gamma counting system consisted of a Canberra model 2022 amplifier and a Canberra model 8075 analog-to-digital converter. The measured spectra were evaluated using Genie 2000 v2.1 software. The gamma measurement times varied between 600 and 5200 s. All gamma spectrometric measurements were performed at fixed geometries (i.e. relative measurements to the original starting material before the separation). For the chemical recovery measurement by gamma spectrometry, the 185.7 keV gamma peak of ^235^U (with an emission probability of 57.2 %) was used. In order to calculate the separation factor of the first separation by gamma spectrometry, the gamma peak at 63.3 keV (emission probability of 3.7 %) of the short-lived ^234^Th (*T*_1/2_ = 24.1 days) was used. Calculation of the Th separation factor via the ^231^Th gamma peak at 25.6 keV resulted in a higher uncertainty due to higher ingrowth correction because of its shorter half-life (*T*_1/2_ = 25.5 h). The time elapsed between the separation and gamma measurement was registered in each case. Background was measured every measurement day.

For the age calculations the ^234^U to ^230^Th half-lives reported by Cheng et al. in 2000 were used [(245,250 ± 490) years and (75,690 ± 230) years (*k* = 2), respectively] [29].

All dilutions were done gravimetrically. The solution weights were obtained as the difference of the weight of the sample in the measurement vials and the tare vial weights for each sample step. The overall uncertainties were calculated taking into account the uncertainty of the weight measurements, tracer concentrations, measured isotope ratios, relative atomic masses and half-lives according to ISO/BIPM guide [33]. The given uncertainties in the present work are expanded uncertainties with a coverage factor of *k* = 2 if not indicated otherwise. The U chemical recovery and Th separation factor calculations were carried out by Excel^®^, while for the age calculations commercially available software, GUM Workbench was used [34].

### Development and test separation for the CRM production

For the preparation of the age dating material several separation steps were required due to the exceptionally large amount of uranium (at least 5 grams as final product) together with a very high Th separation factor of at least 1.76 × 10^4^. Out of the possible separation techniques extraction chromatographic separation was chosen as the best option with respect to purity, separation efficiency and rapidity [35], and TEVA resin was selected due to its high Th retention and capacity besides the low U retention. Silica gel, which has a very high Pa adsorption efficiency, was also layered on the top of the TEVA resin to facilitate Pa removal. Based on the reported characteristics of the TEVA resin, the maximum U load on the column has to be less than 400 mg with a U concentration of 40 mg/mL in order to avoid Th bleeding from the column [35]. The nitric acid concentration was 2–3 M, where the Th retention is close to maximum on the TEVA resin.

**Fig. 1:**
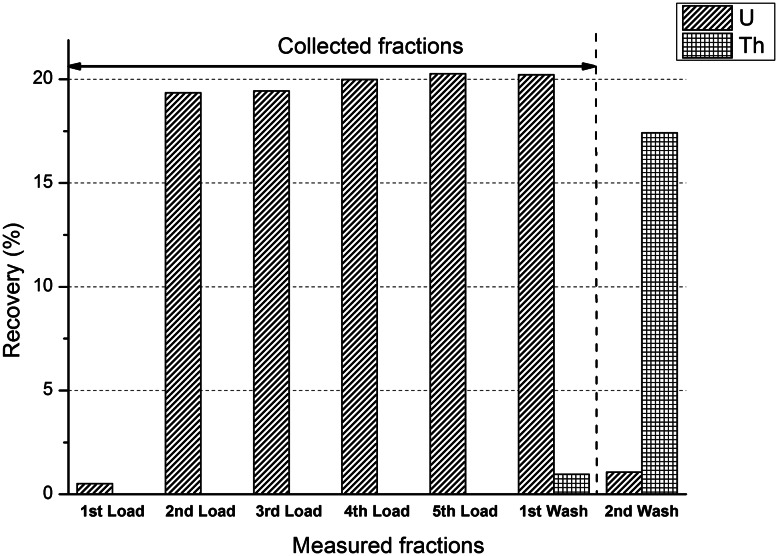
The U and Th elution profile on the silica gel/TEVA column

In order to test the proposed methodology and to measure the Th separation efficiency and U chemical recovery, an aliquot of the feed solution containing 400 mg of U was subjected to the column separation on the silica gel/TEVA column. The volume of the load was 10 mL (40 mg U mL^−1^), which was added in 2 mL portions, followed by twice 2 mL 2 M HNO_3_ wash. The first 2 mL HNO_3_ was used also to rinse the sample vial. The flow rate was approximately 5 min mL^−1^. The fractions were separately collected after each solution addition and were measured by gamma spectrometry. The profile of the U and Th elution on the TEVA/silica gel column is shown in Fig. 1.

Based on the results approximately 99 % of uranium could be recovered if the load fractions and the first 2 mL wash are collected, with an approximate Th separation factor of 96. This means that for four consecutive separation steps a cumulative Th separation factor of approximately 96^4^ = 8.6 × 10^7^ is expected, which is sufficiently high to decisively reach the target separation factor. For such a four-step separation scheme a total U recovery of (99 %)^4^ = 96 % is expected. Therefore, to guarantee sufficient reserve to compensate for the possible losses during the CRM production, it was decided to use a total amount of 6 grams uranium for the separation.

The sample feed aliquot containing about 6 grams of uranium had to be distributed on several columns to avoid overloading and deterioration of the Th separation factor or the uranium recovery. In order not to exceed 400 mg U load on each column, the feed solution was divided into 16 aliquots and loaded on 16 separate extraction chromatography columns simultaneously.

### Preparation of the reference material

**Fig. 2:**
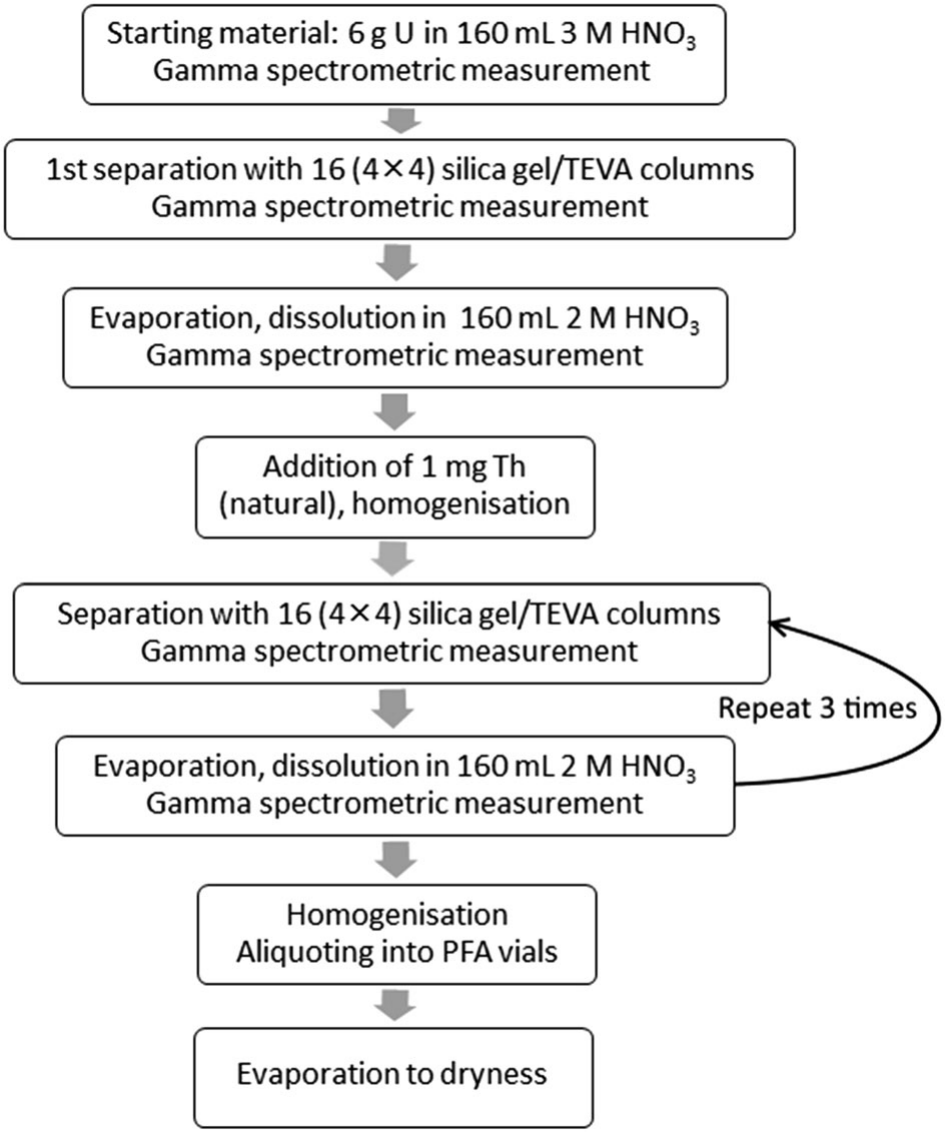
Flow chart of the CRM production

An aliquot of the low-enriched uranium feed solution containing approximately 6 g of uranium was used for the production of the reference material. The solution was diluted to 160 mL 3 M HNO_3_ in a glass beaker, which served as the feed solution for the first separation. A 1.000 mL aliquot of the feed solution was measured by gamma spectrometry for the U recovery and Th separation factor calculations. In the forthcoming steps all gamma measurements were carried out with the same geometry, thus the obtained results relate to the original starting material solution. After placing back the aliquot used for gamma measurement, the solution was loaded simultaneously on 16 silica gel/TEVA columns. The loads and the 2 mL 2 M HNO_3_ wash solutions from the columns were collected in four 150-mL glass beakers, i.e. each glass beaker collected the solutions from four columns. After thorough mixing, all four solutions were measured by gamma spectrometry and checked for the appropriate U recovery and Th separation factors. The solutions used for gamma spectrometry were returned to the original samples, then the solutions in the four glass beakers were mixed together in a 250-mL glass beaker, followed by twice 2 mL 2 M HNO_3_ rinsing for each beaker. The solution was thoroughly homogenized. Gamma spectrometry measurement was performed on a 1.000 mL aliquot, which served to calculate the U recovery and Th separation factor for the first separation step. The first separation was accomplished on 3 July, 2012. After returning the aliquot used for gamma spectrometry, the solution was slowly evaporated overnight. The next day, the solid residue was dissolved in 160 mL 2 M HNO_3_ while heating gently on a hot-plate. After cooling and weight measurement, gamma spectrometry measurement was performed on a 1.000 mL aliquot. However, as the ingrowth of ^234^Th is slow, it is difficult to measure the Th separation factor by gamma spectrometry effectively in freshly separated uranium samples. Therefore, the Th separation factor for the next three steps was also determined by the addition of a high amount of natural Th to the sample after the first separation and its re-measurement from the final product. Therefore, 1 mL of 1000 μg g^−1^ natural Th solution (1 mg Th) was added to the sample after the first separation step. After mixing, the chemical separation was repeated another three times as described above, with the exception that no further natural Th was added to the material (Fig. 2). Gamma spectrometry was performed before and after each separation step.

The final, fourth separation was carried out on 9 July 2012. This date corresponds to the production date of the CRM. The total length of the final separation lasted 176 min (about 3 h), the median of the time of the start and the finish of the last separation was 11:08 a.m. Therefore, the uncertainty of the production date intrinsic to the exact time of the last chemical separation was estimated to be 90 min. This uncertainty has been taken into account for the combined uncertainty calculation of the final certified production date.

The final purified solution was thoroughly homogenized and aliquoted into pre-cleaned perflouroalkoxy alkane (PFA) screw-cap vials right after the homogenization to avoid possible loss by adsorption. The samples were gently evaporated to dryness on hotplate at about 90 °C, capped, labelled and stored at EC-JRC-ITU before shipment to EC-JRC-IRMM. The evaporation was gentle and resulted in a uranyl nitrate/UO_3_ form as the final product. Finally, based on the U recovery measured by the gamma spectrometry, 108 units of 20-mg U items (IRMM-1000a) and 53 units of 50-mg U items (IRMM-1000b) were produced. The flow chart of the CRM production is shown in Fig. 2. Aliquots of the remaining purified sample solution were also used to perform the U isotopic analyses and concentration measurements, impurity analyses and age dating (^230^Th/^234^U measurement) by ICP-MS and TIMS.

## Description of the final product

### Uranium chemical recovery

Based on the gamma measurement the uranium chemical recovery was measured to be (83.7 ± 0.6) %, slightly lower than expected from the test measurement (about 95 % in each step). The lower recovery may be due to the successive separations with multiple aliquotings and evaporation steps compared to the test separation. By all means, the amount of purified uranium (4.98 g) was appropriate for the project purposes, sufficient to produce the envisaged number of units in two unit sizes.

### Th separation factor

The Th separation efficiency, which is the vital element to make sure that no residual ^230^Th remain after the preparation and the ^230^Th/^234^U ratio is determined only by the ^234^U decay (Eq. 2), was verified by three methods: (i) using gamma spectrometry and measuring the separation efficiency after each separation step; (ii) by the addition of ^232^Th to the material at high amount after the first separation and its re-measurement from the final product, and (iii) by measuring the ^230^Th/^234^U (i.e. age) from the freshly produced final product.

The Th separation factors measured by gamma spectrometry were higher than 714, 22, 24 and 75 in the first, second, third and fourth steps, respectively. This resulted in a cumulative Th separation factor of higher than 2.8 × 10^7^ considering all the four steps. Knowing the initial ^230^Th/^234^U ratio of 3.40 × 10^−5^ (reference date: 12 July 2012) from the feed solution, this separation factor corresponds to a maximum bias from the residual ^230^Th of 1.3 h.

By the addition of ^232^Th to the material after the first separation step and its re-measurement from the final product, the cumulative Th separation factor for the 2nd to 4th separations could be obtained. Using the routine measurement procedure applied for impurity analysis at JRC-ITU [32], the Th concentration in the final product was measured to be less than 0.01 μg Th g^−1^ U, corresponding to a Th separation factor higher than 2.54 × 10^4^, equivalent to about 4.1 h’ bias. This shows that even the last three separation steps had been sufficient to achieve the target residual ^230^Th corresponding to less than 6 h’ bias.

The freshly purified solution was also measured by ICP-MS 3.0 days after the reference material production. The ^230^Th was pre-concentrated, separated and measured using the EC-JRC-ITU procedure [27]. The ^230^Th concentration in the purified final solution was below the detection limit of 3.2 pg g^−1^ U, corresponding to a model age of less than 7.6 days based on the ^230^Th/^234^U ratio, which is in agreement with the age of the freshly purified sample, and thereby confirms the high Th separation efficiency. Using the chemical separation an improved detection limit could be obtained for ^232^Th (i.e. the dominant component of the natural Th) compared to the routine measurement procedure used for impurity analysis. The measured ^232^Th concentration in the final purified solution was also below the detection limit of 36 ng g^−1^ U, which can be converted to a separation factor higher than 7.1 × 10^6^ for the 2nd–4th separation steps.

The Th separation factors obtained by the three different approaches agree well and confirm the completeness of the Th separation, i.e. the residual ^230^Th in the purified CRM corresponds to a bias of less than 1.3 h. This bias, although a small constituent, has to be taken into account for the final combined uncertainty of the certified production date.

### Impurities in the final reference material

The most significant impurities (above 100 μg g^−1^ U concentration) are Al, Ca, Fe, P and Er with concentrations of 4304 ± 516, 2378 ± 285, 1028 ± 123, 458 ± 55 and 246 ± 30 μg g^−1^ U, respectively. The total impurity content (including most metallic and non-metallic impurities) in the material measured by ICP-MS was less than 9000 μg g^−1^ U, thus no adverse effect is expected for the chemical separations.

### Uranium isotopics before and after preparation

The U isotopic composition of the final reference material was measured using the high-precision MTE-TIMS method [31] and compared with the U isotopics of the starting material to verify that no uranium contamination from a different source (with the possible addition of its Th decay product as well) occurred during the CRM production. The uranium isotopic composition of the final CRM as isotope mass fraction is ^234^U: 0.028553 ± 0.000040, ^235^U: 3.6072 ± 0.0037, ^236^U: 0.090030 ± 0.000082 and ^238^U: 96.274 ± 0.040, which is in complete agreement with the U isotopics in the starting material (“Starting uranium material” Section) even for the minor uranium isotopes, thereby showing that no U contamination occurred during the CRM production.

## Conclusions

A novel age dating certified reference material based on the ^230^Th/^234^U chronometer was prepared in compliance with ISO Guide 34:2009 [25]. The unique methodology is based on the complete and verified separation of the Th decay products at a well-known time, thus it is not necessary to rely on archive results or consensus values (e.g. from inter-laboratory comparison) to derive the production date (age) of the material. Altogether 108 units of 20 mg U (IRMM-1000a) and 53 units of 50 mg U (IRMM-1000b) were produced. The production date for the IRMM-1000a and IRMM-1000b is 9 July, 2012. The completeness of the Th chemical separation was assessed by means of gamma-spectrometry and ICP-MS measurements using three different approaches, and a Th separation factor of higher than 2.8 × 10^7^ was obtained, corresponding to a systematic bias from residual ^230^Th of less than 1.3 h (80 min). The systematic bias related to the finite length of the chemical separation was estimated to be 90 min. The contribution from these biases is very small, and is well below the current analytical capabilities for age dating.

As the ^230^Th/^234^U ratio is dominantly determined by the radioactive decay laws in the presence of the very tiny amount of residual ^230^Th at the time of preparation, the material can also serve as a primary reference material for this ratio. The expected high efficiency Pa separation gives an indication that the certified production dates based on both chronometers agree within uncertainty, but further studies are needed to verify this assumption. The overall certification process will be described in the complementary second part of this paper.
